# From oral dysbiosis to colorectal cancer: reframing fusobacterium nucleatum as an oxidative stress-primed oncobion

**DOI:** 10.3389/fcimb.2026.1904912

**Published:** 2026-08-28

**Authors:** Giordana Di Mauro, Giuliana Ciappina, Vincenzo Quagliariello, Patrizia Carroccio, Pierluigi Consolo, Teresa Pollicino, Nicola Maurea, Antonio Ieni, Valeria Zuccalà, Carmen Simone, Alessandro Ottaiano, Salvatore Berretta, Teresa Maltese, Massimiliano Berretta

**Affiliations:** 1 School of Specialization in Medical Oncology, University of Messina, Messina, Italy; 2 Translational Molecular Medicine and Surgery, Department of Clinical and Experimental Medicine, University of Messina, Messina, Italy; 3 Department of Medical Sciences, University of Ferrara, Ferrara, Italy; 4 Applied Biology and Experimental Medicine, Department of Chemical, Biological, Pharmaceutical and Environmental Sciences, University of Messina, Messina, Italy; 5 Division of Cardiology, Istituto Nazionale Tumori-IRCCS-Fondazione G. Pascale, Napoli, Italy; 6 Department of Clinical and Experimental Medicine, University of Messina, Messina, Italy; 7 Department of Human Pathology of Adults and Developmental Age “Gaetano Barresi,” Division of Pathology, University of Messina, Messina, Italy; 8 Division of Medical Oncology, “G. Martino” University Hospital, University of Messina, Messina, Italy; 9 Division of Innovative Therapies for Abdominal Metastases, Istituto Nazionale Tumori IRCCS Fondazione G. Pascale, Naples, Italy

**Keywords:** chemoresistance, colorectal cancer, Fusobacterium nucleatum, immune evasion, microbiota, oncobiont, oxidative stress, precision oncology

## 1 Introduction

Colorectal cancer (CRC) remains the third most common malignancy and the second leading cause of cancer-related mortality worldwide, with increasing incidence especially in individuals under the age of 50 (Siegel et al., 2019). Despite advances in surgical techniques, chemotherapy, and the selective benefit of immune checkpoint inhibitors (ICIs), outcomes for most patients remain suboptimal (Morris et al., 2023). This clinical reality has intensified the search for novel biological factors that drive CRC progression and resistance, as well as predictive biomarkers and therapeutic targets that operate beyond traditional mutational profiles. It has been revealed that the initiation and progression of CRC is instigated by genetic factors and environmental factors, among which the gut microbiota is a special environmental risk factor. The gut microbiota has become a central player in colorectal carcinogenesis. A growing body of evidence now supports the concept that specific bacterial species not only passively colonize tumor tissue but actively drive tumor initiation, progression, immune evasion, and drug resistance (Zitvogel et al., 2017; Fan et al., 2021; Qu et al., 2023). Among these, Fusobacterium nucleatum (F. nucleatum) has attracted exceptional scientific attention. Early genomic studies by Kostic et al. (2013) and Castellarin et al. (2012) independently demonstrated that F. nucleatum was significantly more abundant in CRC tissue than in adjacent normal mucosa. F. nucleatum is a Gram-negative, obligately anaerobic, spindle-shaped bacterium belonging to the family Fusobacteriaceae, occupying a key structural position in oral biofilms (Kaplan et al., 2009; Hofer, 2022). Under conditions of dysbiosis caused by factors such as diet, smoking, oxidative stress, and antibiotic exposure, F. nucleatum transitions from commensal to pathobiont, expanding beyond its oral niche and disseminating systemically (Han, 2015; Abed et al., 2020). Genomic evidence from matched patient samples indicates that strains isolated from colorectal tumors are virtually identical to those recovered from the oral cavity of the same individual, supporting a clonal oral origin for intestinal colonization (Komiya et al., 2019). In CRC, F. nucleatum promotes carcinogenesis through multiple converging pathways: it activates the Wnt/β-catenin signaling axis via its adhesin FadA; induces chronic inflammation mediated by the NLRP3 inflammasome; upregulates oncogenic microRNAs; modulates the tumor immune microenvironment; and confers resistance to conventional chemotherapy through autophagy-dependent mechanisms (Rubinstein et al., 2013; Yang et al., 2017; Yu et al., 2017). Importantly, F. nucleatum abundance correlates with tumor stage and is a robust predictor of reduced disease-free and overall survival (Tahara et al., 2014; Kim et al., 2022; Wang et al., 2025).

Here, we critically examine the existing evidence to offer a comprehensive and integrated perspective on F. nucleatum, exploring its role as an oncobiont that actively drives tumor progression, its value as a prognostic stratifier, and its potential as an emerging target for microbiota-informed precision oncology.

Although Fusobacterium nucleatum is widely recognized as a colorectal cancer-associated oncobiont, current models still inadequately explain the biological transition from oral commensalism to colorectal pathogenicity. We propose that oxidative stress within the oral microenvironment represents the priming event that activates the pathogenic program of F. nucleatum prior to intestinal dissemination.

Rather than providing a purely systematic overview, this opinion paper proposes a translational perspective in which oxidative stress acts as a critical ecological trigger capable of reshaping *F. nucleatum* pathogenicity and promoting inflammation-associated colorectal carcinogenesis. Within this framework, oxidative adaptation is interpreted as a dynamic contributor to bacterial persistence, tumor colonization, and therapy resistance.

## 2 Background: what we already know

Historically, *F. nucleatum* was classified into four subspecies (Wolf et al., 2025), although recent genomic analyses demonstrated that these lineages represent distinct species (Krieger et al., 2024). Among them, *F. animalis* is particularly enriched in CRC tissue (Zepeda-Rivera et al., 2024). A landmark *Nature* study identified two major phylogenetic clades within *F. animalis*, C1 and C2, with C2 emerging as the dominant CRC-associated lineage, while C1 was later reclassified as *F. watanabei* (Zepeda-Rivera et al., 2024; Sivertsen et al., 2025). Importantly, the C2 clade exhibits enhanced acid resistance, adhesin expression, and tumor-colonizing capacity, promoting colorectal adenoma formation in murine models (Zepeda-Rivera et al., 2024; Capuozzo et al., 2025). These findings highlight the strain-specific pathogenic potential of the *F. nucleatum* complex and support the need for precision diagnostic and therapeutic approaches.

*F. nucleatum* reaches the colorectal mucosa mainly through the oro-intestinal axis, either via gastrointestinal transit or hematogenous dissemination from the oral cavity (Abed et al., 2020). Genomic studies confirmed that oral and tumor-associated strains are often genetically identical, supporting the oral cavity as the primary reservoir for CRC colonization (Komiya et al., 2019). Although rarely detected in healthy colonic microbiota, *F. nucleatum* is consistently enriched in CRC tissue (Castellarin et al., 2012; Nawab et al., 2023).

Three major adhesins mediate colonization and tissue tropism. FadA binds E-cadherin, activating β-catenin signaling and NF-κB inflammatory pathways while facilitating endothelial disruption and systemic dissemination (Fardini et al., 2011; Su et al., 2020). Fap2 mediates selective binding to tumor-associated Gal-GalNAc glycans and suppresses NK and T-cell activity through TIGIT interaction (Gur et al., 2015; Abed et al., 2016; Schöpf et al., 2025). Importantly, although direct mechanistic evidence remains limited, Fap2 interaction with FN1 deposited within the extracellular matrix may stabilize tumor colonization and immune evasion. Finally RadD contributes to biofilm organization and inhibits NK-cell-mediated tumor killing through Siglec-7 binding (Galaski et al., 2024; Quagliariello et al., 2025), while also enhancing CRC colonization *in vivo* (Zhang et al., 2024). Together, these findings indicate that tumor tropism and immune escape depend on coordinated adhesin activity rather than FadA alone, supporting the need for multi-target therapeutic strategies (Incognito et al., 2025).

One of the most intriguing advances in *F. nucleatum* research concerns the role of oxidative stress in driving the transition from commensal organism to oncogenic pathobiont. Scano et al. (2025) developed an *in vitro* “Fusobacterium stress chamber” exposing clinical strains to sub-inhibitory H_2_O_2_ concentrations mimicking oral dysbiosis. Oxidative preconditioning significantly increased biofilm formation, dimethyl sulfide (DMS) production, and HT-29 colonization, with approximately doubled invasive capacity compared to unstressed strains (Scano et al., 2025). These findings suggest that oxidative stress directly enhances bacterial virulence before intestinal dissemination.

Potential triggers of oral oxidative stress include pro-inflammatory diets, cigarette smoke (Zieba et al., 2024), and H_2_O_2_-containing antiseptic agents (Scano et al., 2025). Under these conditions, *F. nucleatum* acquires a more invasive phenotype characterized by increased adhesin expression, enhanced oxidative adaptation, and modulation of host inflammatory pathways.

Importantly, oxidatively activated *F. nucleatum* increases pri-miR-155 while suppressing pri-miR-146a, promoting inflammation, tumor progression, and chemoresistance (Garo et al., 2021; Bostanshirin et al., 2023; Wu et al., 2023; Scano et al., 2025). Simultaneously, ALDH2 upregulation may reduce intracellular malondialdehyde (MDA) accumulation, protecting CRC cells from oxidative cytotoxicity and further contributing to therapy resistance (Muro et al., 2024; Scano et al., 2025).

Lastly Dadashi et al. (2021) demonstrated that the recombinant FadA adhesin from *Fusobacterium nucleatum* significantly promotes the proliferation of SW480 colorectal cancer cells in a dose- and time-dependent manner. These findings suggest that FadA may directly contribute to colorectal cancer progression by enhancing tumor cell growth, further supporting the involvement of *F. nucleatum* in colorectal carcinogenesis (Dadashi et al., 2021) Collectively, these findings support the hypothesis that oxidative stress acts as a biological priming event transforming *F. nucleatum* into an aggressive oncobiont before colorectal colonization.

## 3 Oxidative stress, *Fusobacterium nucleatum* and molecular mechanisms of tumor promotion and chemo-resistance

Over the past two years, our research group has increasingly focused on elucidating the mechanisms through which *Fusobacterium nucleatum* promotes inflammation and contributes to the establishment of a pro-tumorigenic microenvironment. Particular attention has been devoted to the interplay between oxidative stress, inflammatory signaling, microbial colonization, and immune dysregulation in colorectal cancer and inflammatory bowel disease. The following sections summarize the main mechanistic and translational evidence currently supporting the emerging role of *F. nucleatum* in inflammation-driven colorectal carcinogenesis.

As previously discussed, the adhesin FadA plays a central role in *F. nucleatum*-mediated colorectal carcinogenesis. In particular, FadA binding to E-cadherin activates β-catenin signaling and promotes pro-inflammatory and oncogenic pathways involved in tumor progression. The FadA/E-cadherin interaction activates β-catenin signaling, inducing transcription of oncogenic targets including cyclin D1, c-Myc, and MMP7 (Rubinstein et al., 2013; Mondal et al., 2025). This pathway promotes epithelial proliferation, cancer stem-cell phenotypes, and links chronic inflammation, particularly in inflammatory bowel disease (IBD), with increased CRC risk (Kostic et al., 2013; Rubinstein et al., 2013; Mondal et al., 2025). FadA also contributes to genomic instability through Chk2 upregulation (Consolo et al., 2025).

In parallel, *F. nucleatum* activates the NLRP3 inflammasome, leading to caspase-1 activation and secretion of IL-1β and IL-18 (Ahechu et al., 2018). These cytokines sustain a pro-tumorigenic inflammatory microenvironment characterized by angiogenesis, epithelial–mesenchymal transition (EMT), immune evasion, and myeloid-cell recruitment (Ahechu et al., 2018; Consolo et al., 2025). NLRP3 therefore emerges as a promising therapeutic target, particularly given the efficacy of novel aryl-sulfonamide inhibitors in preclinical models (Albanese et al., 2023).

Lipopolysaccharide (LPS)-mediated TLR2/TLR4–MyD88 activation further stimulates NF-κB and MAPK signaling, inflammatory cytokine production, anti-apoptotic proteins, EMT, autophagy, and resistance to 5-fluorouracil and oxaliplatin (Kostic et al., 2013; Yang et al., 2017; Yu et al., 2017). The same pathway promotes miR-21 upregulation and iron-dependent secretion of CCL8 and CXCL5, mechanisms associated with invasion, macrophage activation, and poor prognosis (Yang et al., 2017; Ciappina et al., 2025– Wang and Fang, 2023).

*F. nucleatum* reshapes the CRC miRNA landscape through induction of oncogenic miRNAs, including miR-31 and oxidative stress-associated miR-155, while suppressing anti-inflammatory regulators such as miR-146a (Xu et al., 2021; Xu et al., 2022; Tang et al., 2023; Zhang et al., 2023). These alterations promote epithelial–mesenchymal transition (EMT), extracellular matrix degradation, metastatic dissemination, and resistance to apoptosis (Ma et al., 2024). Consistently, identical *F. nucleatum* strains have been identified in primary CRC lesions and liver metastases, supporting bacterial co-migration during metastatic spread (Shigematsu et al., 2024; Ye et al., 2024).

Importantly, *F. nucleatum* contributes to chemoresistance through interconnected inflammatory, autophagic, and redox-dependent pathways. TLR4/MyD88-mediated autophagy reduces sensitivity to 5-fluorouracil, while the E-cadherin/β-catenin/GPX4 axis suppresses ferroptosis and promotes oxaliplatin resistance (Li et al., 2024; Ciappina et al., 2025). BIRC3 overexpression and exosome-mediated transfer of resistance-associated molecules further amplify anti-apoptotic and chemoresistant phenotypes (Zhang et al., 2019; Hui et al., 2024). Collectively, these mechanisms contribute to metastatic progression, systemic inflammation, and poor clinical outcome, consistent with the correlation between fecal *F. nucleatum* levels and circulating NET markers (Kong et al., 2023). The main biological mechanisms through which *Fusobacterium nucleatum* contributes to colorectal carcinogenesis are summarized in **Table 1**.

**Table 1:** Biological mechanisms linking Fusobacterium nucleatum to colorectal carcinogenesis.

Mechanism/molecular target	Biological function	Impact on CRC progression	Strength of evidence	Potential clinical implications
FadA – E-cadherin interaction	Activation of β-catenin and NF-κB signaling	Tumor initiation, proliferation, stemness, inflammation	Strong	Therapeutic target; biomarker of aggressive disease
Fap2 – Gal-GalNAc binding	Selective recognition of tumor cells	Tumor colonization and persistence	Strong	Target for anti-adhesion strategies and microbial eradication
Fap2 – TIGIT interaction	Suppression of NK and T-cell activity	Immune escape and reduced antitumor immunity	Strong	Potential synergy with immunotherapy
RadD – Siglec-7 interaction	Inhibition of NK-cell cytotoxicity	Immune evasion and tumor persistence	Moderate to Strong	Emerging immunomodulatory target
NLRP3 inflammasome activation	IL-1β and IL-18 secretion, chronic inflammation	EMT, angiogenesis, tumor-promoting inflammation	Moderate to Strong	Potential use of NLRP3 inhibitors
TLR4/MyD88 signaling	NF-κB activation, cytokine production, autophagy induction	Tumor growth and chemoresistance	Strong	Biomarker of resistance; therapeutic target
miR-21 induction	Promotion of invasion and survival pathways	Tumor progression and poor prognosis	Strong	Prognostic biomarker
miR-31 induction	Enhancement of tumorigenic signaling	CRC progression and metastasis	Moderate	Biomarker candidate
miR-155 upregulation	Oxidative stress-associated inflammatory reprogramming	Inflammation, tumor progression, chemoresistance	Moderate	Potential biomarker of oxidative priming
miR-146a suppression	Loss of anti-inflammatory regulation	Persistent inflammation and carcinogenesis	Moderate	Marker of inflammatory dysregulation
Autophagy activation	Cellular survival under chemotherapy	5-FU resistance	Strong	Predictor of treatment response
GPX4-mediated ferroptosis suppression	Protection from oxidative cell death	Oxaliplatin resistance	Moderate to Strong	Therapeutic vulnerability
Oxidative stress priming of F. nucleatum	Increased invasiveness, biofilm formation and colonization capacity	Transition from commensal to pathobiont	Preliminary but biologically plausible	Candidate preventive and therapeutic target
Fap2–FN1 interaction	Putative stabilization of bacterial docking within tumor ECM	Tumor persistence and metastatic dissemination	Hypothesis-generating/Limited direct evidence	Requires experimental validation before translation
Oral–intestinal dissemination axis	Migration of oral strains to colorectal tissue	Establishment of tumor-associated colonization	Strong	Basis for salivary and fecal screening approaches

## 4 *Fusobacterium nucleatum* and the emerging paradigm of microbiome-driven precision oncology: a conceptual perspective

The growing body of evidence linking *Fusobacterium nucleatum* to CRC and IBD suggests that this bacterium may represent more than a passive microbial biomarker of disease. Rather, *F. nucleatum* increasingly appears to function as a biologically active oncobiont capable of orchestrating chronic inflammation, epithelial transformation, immune escape, metastatic dissemination, and resistance to therapy through interconnected oxidative and inflammatory pathways (Kostic et al., 2013; Yang et al., 2017; Yu et al., 2017; Capuozzo et al., 2025).

This concept is particularly relevant in chronic inflammatory disorders such as IBD, where persistent mucosal inflammation, epithelial barrier disruption, and oxidative stress create a permissive environment for bacterial colonization and tumorigenesis. Several studies have demonstrated that *F. nucleatum* abundance is significantly increased in both CRC and IBD tissues compared to healthy controls, supporting the existence of a shared pathogenic axis linking chronic inflammation and colorectal carcinogenesis (Strauss et al., 2011; Capuozzo et al., 2025).

We argue that oxidative stress may represent the central mechanistic bridge connecting oral dysbiosis, chronic intestinal inflammation, neoplastic transformation, metastatic progression, and chemoresistance in *F. nucleatum*-associated disease. In this model, reactive oxygen species (ROS) generated by smoking, pro-inflammatory diets, periodontal inflammation, and intestinal inflammatory activity do not merely damage host tissues but actively reshape bacterial behavior, promoting the transition of *F. nucleatum* from commensal organism to aggressive pathobiont (Zieba et al., 2024; Scano et al., 2025).

Experimental evidence strongly supports this hypothesis. As discussed above, oxidative stress appears to play a central role in the pathogenic reprogramming of *F. nucleatum*. In support of this hypothesis, Scano et al. (2025) demonstrated that H_2_O_2_ exposure enhances bacterial biofilm formation, invasiveness, dimethyl sulfide production, and colonization of HT-29 cells (Garo et al., 2021; Wu et al., 2023; Scano et al., 2025).

In our view, these findings support a broader biological paradigm in which oxidative stress acts as a “priming force” that enables *F. nucleatum* to acquire invasive, pro-inflammatory, and tumor-promoting capabilities before intestinal dissemination. Once established within the colorectal microenvironment, the bacterium further amplifies carcinogenesis through activation of β-catenin signaling, NLRP3 inflammasome activation, NF-κB-mediated cytokine production, and suppression of antitumor immunity (Rubinstein et al., 2013; Ciappina et al., 2025; Consolo et al., 2025). Importantly, these pathways are also deeply interconnected with extracellular matrix remodeling and metastatic progression.

A particularly intriguing and potentially innovative aspect concerns the role of fibronectin 1 (FN1) within the tumor microenvironment. Traditionally considered a structural component of the extracellular matrix, FN1 may instead function as an active regulator of microbial tumor colonization. Emerging evidence suggests that the *F. nucleatum* adhesin Fap2 not only mediates binding to tumor-associated Gal-GalNAc glycans and TIGIT-dependent immune suppression but may also interact with FN1 deposited within CRC tissue, thereby stabilizing bacterial adhesion and persistence (Gur et al., 2015; Abed et al., 2016; Schöpf et al., 2025).

Although direct mechanistic validation remains limited, emerging evidence supports a potential role for FN1 in stabilizing bacterial persistence within the tumor extracellular matrix. We propose that FN1 acts as an “ecological docking platform” facilitating selective bacterial colonization of the tumor niche. In this framework, the extracellular matrix is no longer a passive scaffold but an active participant in host–microbe interaction. The Fap2–FN1 axis may therefore represent a previously underrecognized microbial checkpoint controlling tumor tropism, immune escape, and metastatic dissemination. This hypothesis is particularly compelling because FN1 overexpression is itself associated with epithelial–mesenchymal transition (EMT), invasive behavior, and poor prognosis in CRC, suggesting a synergistic interaction between tumor matrix remodeling and microbial pathogenicity.

Importantly, the biological effects of *F. nucleatum* appear to extend beyond tumor initiation and progression to include resistance to therapy. Multiple studies have demonstrated that *F. nucleatum* promotes resistance to 5-fluorouracil and oxaliplatin through activation of autophagy, suppression of ferroptosis, anti-apoptotic signaling, and oxidative stress modulation (Yu et al., 2017; Zhang et al., 2019; Li et al., 2024; Ciappina et al., 2026). These observations strongly suggest that bacterial colonization may directly influence treatment responsiveness and clinical outcome.

Within this framework, the ongoing prospective translational clinical study proposed by Consolo et al. (2025) assumes particular relevance. The study is designed to evaluate the relationship between *F. nucleatum*, oxidative stress biomarkers, inflammation, and chemoresistance in patients with CRC and IBD, with the aim of identifying biologically actionable pathways capable of guiding personalized therapeutic interventions (Consolo et al., 2025). We believe that this approach may contribute to redefining the role of microbiomes in precision oncology.

Indeed, if *F. nucleatum* actively contributes to tumor progression and resistance mechanisms, targeting the bacterium or its downstream inflammatory pathways could become a clinically meaningful therapeutic strategy. In this regard, NLRP3 inflammasome inhibitors represent particularly promising candidates. Pharmacological blockade of NLRP3 may simultaneously reduce chronic inflammation, reverse tumor-promoting cytokine signaling, and restore sensitivity to chemotherapy (Albanese et al., 2023; Consolo et al., 2025). Likewise, selective antimicrobial approaches directed against *F. nucleatum*, including narrow-spectrum antibiotics, bacteriophages, or adhesin-targeting strategies, may offer opportunities to reduce bacterial-driven carcinogenic signaling while preserving commensal microbiota integrity (Capuozzo et al., 2025; Incognito et al., 2025).

From a preventive perspective, this concept may have even broader implications. If persistent *F. nucleatum* colonization contributes to the progression from chronic inflammation to neoplasia, particularly in high-risk populations such as IBD patients, microbiota-targeted interventions could potentially reduce long-term CRC risk. Although this hypothesis remains speculative and requires prospective validation, it introduces the provocative possibility that modulation of oncogenic bacteria may eventually become part of future cancer prevention strategies.

Ultimately, we propose that the next frontier of precision oncology may not solely depend on tumor genomics, but also on the identification of microbiome-dependent inflammatory and oxidative pathways that shape tumor behavior and therapeutic response. In this context, *F. nucleatum* may represent one of the first clinically actionable microbial targets in colorectal cancer biology.

**Figure 1:**
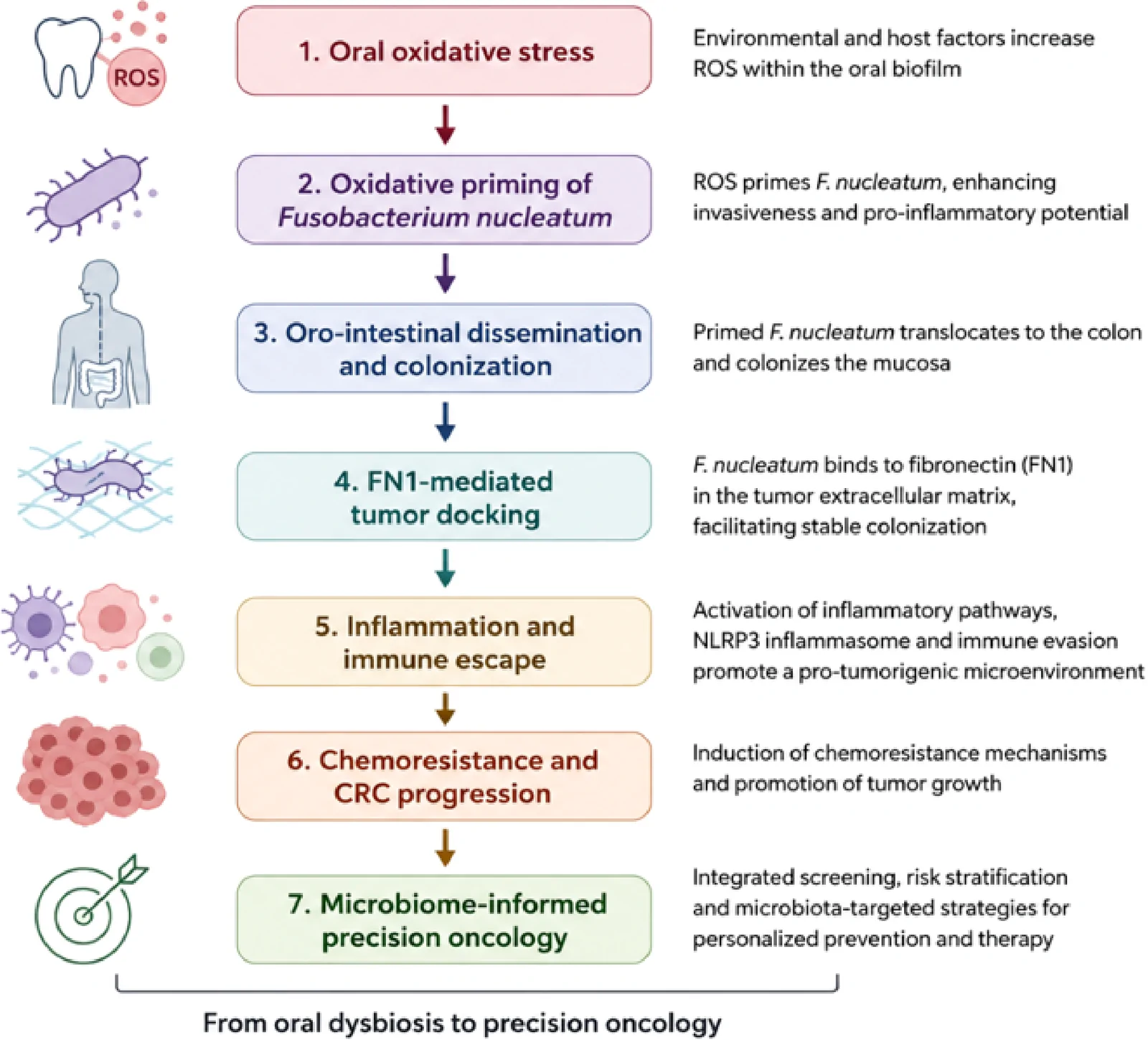
Proposed hypothetical conceptual framework linking oxidative stress adaptation of *Fusobacterium nucleatum* to colorectal carcinogenesis and microbiota-driven precision oncology.

**Figure 1** presents a hypothetical conceptual framework linking oxidative stress adaptation of *Fusobacterium nucleatum* to colorectal carcinogenesis and microbiota-driven precision oncology.

Chronic oral oxidative stress induced by smoking, diet, inflammation, and dysbiosis may promote the transition of *F. nucleatum* from commensal bacterium to pro-oncogenic pathobiont. Oxidative priming enhances bacterial virulence, biofilm formation, and invasive capacity, facilitating oro-intestinal dissemination and selective colonization of colorectal tissue. Within the tumor microenvironment, interaction between bacterial adhesins and extracellular matrix components such as fibronectin 1 (FN1) may stabilize tumor docking and persistence. These processes promote inflammation, immune escape, chemoresistance, and metastatic progression through activation of pathways including NF-κB, β-catenin, and NLRP3 inflammasome signaling. Collectively, this model supports the emerging role of oxidative stress and microbiota adaptation in integrated CRC screening, preventive oncology, and microbiome-informed precision oncology strategies.

## 5 Conclusion

In conclusion the growing evidence linking *Fusobacterium nucleatum* to oxidative stress, chronic inflammation, immune escape, and chemoresistance supports a shift from purely tumor-centered oncology toward a microbiome-informed precision medicine framework (Yu et al., 2017; Zhang et al., 2019; Jiang et al., 2023; Li et al., 2024; Ciappina et al., 2026).

Current CRC screening strategies rely mainly on colonoscopy and fecal immunochemical testing (FIT), but these approaches insufficiently capture the biological mechanisms underlying inflammation-driven carcinogenesis. Combined salivary and fecal microbiota profiling may therefore represent a promising strategy, particularly since tumor-associated *F. nucleatum* strains are often genetically identical to those isolated from the oral cavity of the same patient (Komiya et al., 2019; Abed et al., 2020). Stool-based quantification of *F. nucleatum*, especially when integrated with FIT and molecular biomarkers, already demonstrates encouraging diagnostic performance (Peng et al., 2018; Liu et al., 2021). Additional precision may derive from oxidative stress biomarkers such as MDA and 4-HNE (Muro et al., 2024; Scano et al., 2025), FN1 expression profiling, and identification of high-risk subtypes such as the *F. animalis* C2 clade (Narii et al., 2023; Zepeda-Rivera et al., 2024; Sivertsen et al., 2025). Collectively, these markers may support an integrated screening model capable of identifying high-risk individuals before overt neoplastic transformation occurs.

Beyond diagnosis, *F. nucleatum* may also contribute to therapeutic stratification. Increasing evidence links bacterial colonization to resistance to chemotherapy and immunotherapy through TLR4/MyD88 signaling, autophagy activation, ferroptosis suppression, and modulation of the tumor immune microenvironment (13,

46,57, 58, 62). In particular, patients with elevated intratumoral bacterial burden, oxidative stress activation, or NLRP3 upregulation may represent biologically distinct CRC subsets requiring alternative therapeutic approaches.

Within this framework, microbiota-driven precision oncology could include prediction of oxaliplatin resistance, identification of patients less responsive to immunotherapy, and selection of candidates for microbiota-targeted interventions. Particularly promising are NLRP3-targeted strategies, which may simultaneously reduce inflammation, oxidative stress, and bacterial-driven carcinogenic signaling (Albanese et al., 2023; Consolo et al., 2025). Similarly, microbiota modulation through selective antibiotics, bacteriophages, probiotics, dietary interventions, and adhesin-targeting approaches may help reduce *F. nucleatum*-associated tumor promotion while preserving commensal microbiota balance (Capuozzo et al., 2025; Incognito et al., 2025).

An equally important implication concerns cancer prevention. If oxidative stress acts as the biological trigger converting *F. nucleatum* from commensal bacterium into oncogenic pathobiont, then reduction of chronic oxidative stress may interfere with early carcinogenic activation. In this context, oral oxidative stress reduction may potentially contribute to preventive anti-oncogenic strategies. Smoking cessation, periodontal disease control, antioxidant-rich diets, and modulation of oral dysbiosis may therefore acquire a previously underappreciated role in CRC prevention, particularly in high-risk populations such as patients with IBD or chronic inflammatory disorders (Shigematsu et al., 2024; Scano et al., 2025).

Despite the rapidly expanding literature, important limitations remain. Most studies are heterogeneous in terms of sampling methods, microbiota analysis, and clinical endpoints, while the causal role of *F. nucleatum* in CRC progression still requires prospective validation. Furthermore, most available evidence remains associative, while prospective human studies demonstrating causal relationships between oxidative stress adaptation and CRC progression are still lacking. In this context, the ongoing prospective translational clinical study recently proposed by Consolo et al. (2025) aims to integrate microbiota profiling, cytokine analysis, oxidative stress biomarkers, colonoscopic assessment, and CT-based radiomics in healthy subjects, IBD patients, and CRC patients. Colonoscopies and fecal sample collection are already underway, with the goal of clarifying whether *F. nucleatum* and oxidative stress-related pathways may serve not only as biomarkers of disease progression, but also as actionable targets for microbiota-driven precision oncology. Indeed, radiomics may potentially provide non-invasive surrogate markers associated with microbiota-driven tumor phenotypes.

We argue that the next conceptual advance in microbiota-driven oncology will not derive merely from identifying tumor-associated microbes, but from understanding the environmental signals that convert commensals into oncogenic pathobionts. In this context, oxidative stress may represent the biological switch that transforms F. nucleatum from oral symbiont to colorectal oncobiont.

There is a paucity of validated analytical methods in establishing microbiomebased biomarkers in clinical practice. Thus kitted and validated molecular methods with simple interpretation are much warranted for the clinical implementation of these microbiome based biomarkers.

The future of precision oncology may require simultaneous targeting of tumor genomics, inflammatory signaling, and oncogenic microbiota ecosystems.
